# Antibody Targets and Properties for Complement-Fixation Against the Circumsporozoite Protein in Malaria Immunity

**DOI:** 10.3389/fimmu.2021.775659

**Published:** 2021-12-01

**Authors:** Liriye Kurtovic, Damien R. Drew, Arlene E. Dent, James W. Kazura, James G. Beeson

**Affiliations:** ^1^ Life Sciences, Burnet Institute, Melbourne, VIC, Australia; ^2^ Department of Immunology and Pathology, Monash University, Melbourne, VIC, Australia; ^3^ Center for Global Health and Diseases, Case Western University, Cleveland, OH, United States; ^4^ Department of Microbiology, Monash University, Clayton, VIC, Australia; ^5^ Department of Medicine, The University of Melbourne, Parkville, VIC, Australia

**Keywords:** antibody, circumsporozoite protein (CSP), complement, Plasmodium falcifarum, vaccines, malaria

## Abstract

The *Plasmodium falciparum* circumsporozoite protein (CSP) forms the basis of leading subunit malaria vaccine candidates. However, the mechanisms and specific targets of immunity are poorly defined. Recent findings suggest that antibody-mediated complement-fixation and activation play an important role in immunity. Here, we investigated the regions of CSP targeted by functional complement-fixing antibodies and the antibody properties associated with this activity. We quantified IgG, IgM, and functional complement-fixing antibody responses to different regions of CSP among Kenyan adults naturally exposed to malaria (n=102) and using a series of rabbit vaccination studies. Individuals who acquired functional complement-fixing antibodies had higher IgG, IgM and IgG1 and IgG3 to CSP. Acquired complement-fixing antibodies targeted the N-terminal, central-repeat, and C-terminal regions of CSP, and positive responders had greater antibody breadth compared to those who were negative for complement-fixing antibodies (p<0.05). Using rabbit vaccinations as a model, we confirmed that IgG specific to the central-repeat and non-repeat regions of CSP could effectively fix complement. However, vaccination with near full length CSP in rabbits poorly induced antibodies to the N-terminal region compared to naturally-acquired immunity in humans. Poor induction of N-terminal antibodies was also observed in a vaccination study performed in mice. IgG and IgM to all three regions of CSP play a role in mediating complement-fixation, which has important implications for malaria vaccine development.

## Introduction

*Plasmodium falciparum* malaria is a major health concern and the development of a highly efficacious vaccine against this life-threatening disease is a global priority (1). Vaccine development has proven challenging, partly due to the complex life cycle of the malaria-causing parasite and gaps in our understanding of the immune targets and mechanisms that confer protection against disease (2, 3). Few malaria vaccine candidates have demonstrated protective efficacy in clinical trials, which is often modest and short-lived in malaria endemic populations. The leading RTS,S malaria vaccine was 50% efficacious against malaria over the first 12 months in a phase III pediatric clinical trial, which waned to 36% efficacy over 4 years with a booster vaccination (4). While RTS,S vaccine efficacy is modest, this is the first malaria vaccine the World Health Organization has recommended for use in infants and young children living in regions with moderate to high malaria transmission (5). New insights will be important to inform strategies to increase vaccine efficacy and longevity by building on RTS,S or developing novel malaria vaccines.

The most promising malaria vaccine candidates, including RTS,S, target the *P. falciparum* sporozoite developmental form. Sporozoites are transmitted to humans *via* a female *Anopheles* mosquito where they are deposited in the skin and enter the circulation (6). Sporozoites are then passively carried in the blood and arrest at the liver sinusoid, where they encounter a liver hepatocyte for invasion and development. This journey from the skin to the liver can take up to several hours, providing ample opportunity for the immune system to recognize and attack sporozoites in the skin and circulation (6). A single sporozoite develops into approximately 20,000 merozoites that undergo a continuous cycle of invasion and replication within erythrocytes, which is associated with symptomatic illness. Therefore, sporozoites are an attractive vaccine target to prevent the onset of parasitemia and clinical disease.

**Figure 1:**
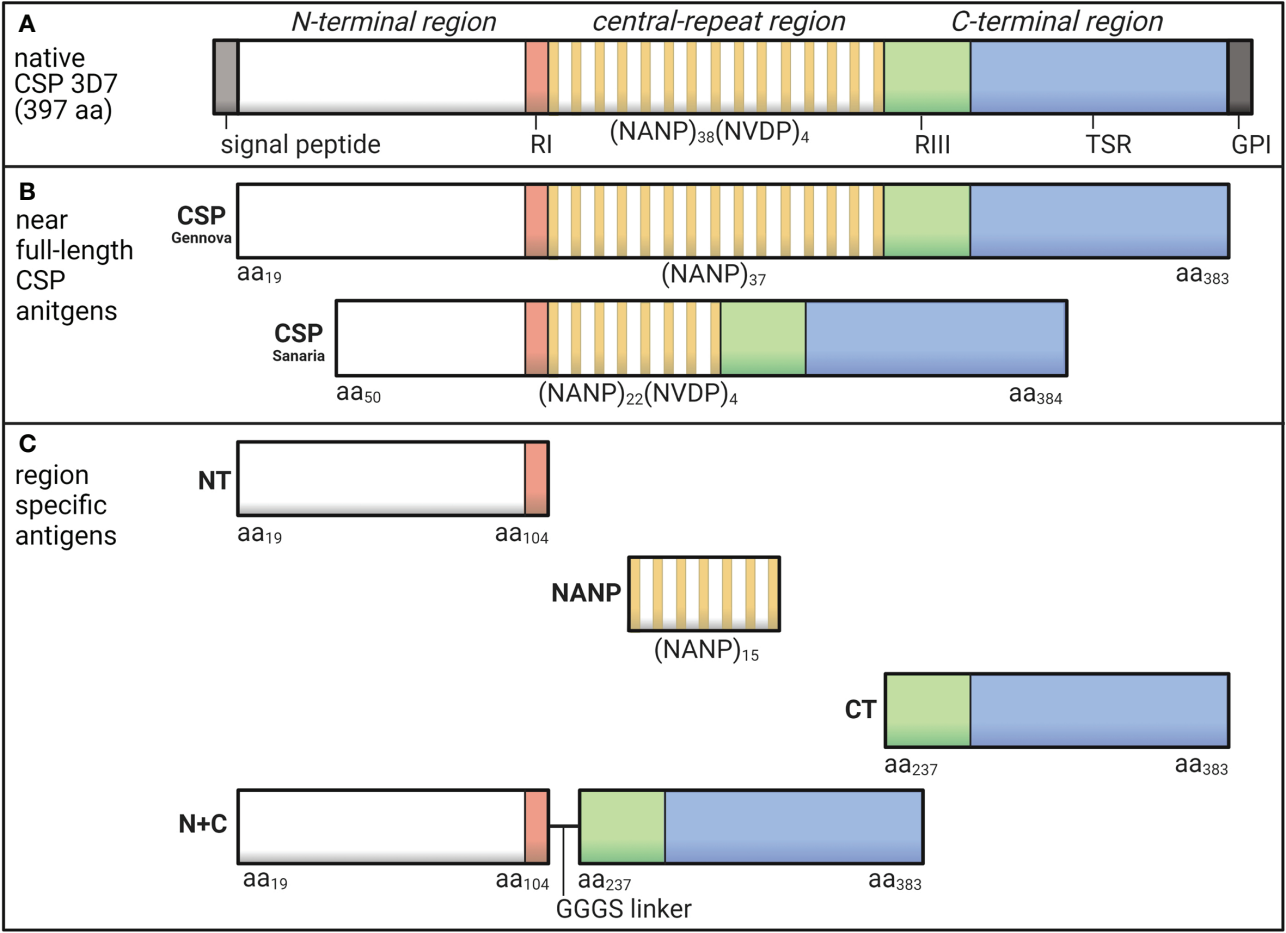
Schematic of *P. falciparum* CSP and region-specific antigens used in this study. **(A)** Native *P. falciparum* CSP (3D7) is 397 amino acids in length and can be divided into the N-terminal region that contains Region I (RI), the central-repeat region largely comprised of NANP tandem repeats, and the C-terminal region that includes Region III (RIII) and the thrombospondin type 1 repeat (TSR) domain. **(B)** In this study, we used two near full-length CSP antigens that lacked the signal peptide and GPI sequences from Gennova (expressed in *E. coli*) and Sanaria (expressed in *P. pastoris*) in human and rabbit studies, respectively. The Gennova CSP contained the major NANP motif repeated 37 times, and the Sanaria CSP began at amino acid 50 and contained the major NANP motif repeated 22 times and the minor NVDP motif repeated 4 times. **(C)** We also used region-specific antigen constructs including a peptide representing the N-terminal region between amino acids 19-104 (NT), a peptide representing the central-repeat region comprised of the NANP motif repeated 15 times (NANP), recombinant protein representing the C-terminal region between amino acids 273-383 (CT) and a truncated form of CSP generated by the N-terminal and C-terminal regions fused together *via* a GGGS short linker (N+C). See **Table 1** for full details. Created using BioRender.com.

The most abundant sporozoite surface antigen is the circumsporozoite protein (CSP), which can be divided into three main regions (**Figure 1A**). The N-terminal region has no predicted structure and contains a sequence known as Region I (RI) (7). The central-repeat region is largely unstructured and entirely comprised of tandem repeats including approximately 38 NANP repeats, although the number of repeats can vary between isolates (8), and few NVDP minor repeats (7, 9). The C-terminal region has a unique tertiary structure formed by two disulfide bonds and contains the GPI anchor site and a thrombospondin type 1 repeat (TSR), which is found in other proteins and is known for its adhesive properties (10, 11). The partly flexible nature of CSP allows the N-terminal region to partially mask the TSR, which is a favorable conformation for sporozoite migration in the skin and arrestment at the liver (12). Studies using mouse and *in vitro* models found that when in contact with a hepatocyte, RI is cleaved and the TSR is unmasked, which appears important for hepatocyte invasion to occur (13). The different regions of CSP each play a unique role and may be exposed or partially masked at different stages of sporozoite infection.

RTS,S and the similar R21 vaccine are based on a truncated form of CSP that lacks the N-terminal region and NVDP minor repeats (14, 15). These vaccines confer modest protection against malaria in clinical trials and vaccine efficacy has some association with anti-CSP antibody titer (15–17). However, the specific targets and mechanisms of antibodies that confer protective immunity are poorly understood, which hinders the development of novel and next-generation malaria vaccines with improved immunogenicity and efficacy (1). There is also a need for greater evaluation of functional antibody activities induced by candidate vaccines (18). There has been a significant focus on the role of antibodies that directly inhibit sporozoite invasion of hepatocytes (19). *In vivo*, such a mechanism would occur towards the end of the liver-stage of infection that occurs in less than a minute, representing only a short window of opportunity for immune attack relative to the amount of time a sporozoite is exposed to the immune system. Furthermore, it has not yet been demonstrated that these invasion-inhibitory antibodies correlate with vaccine efficacy (20). Antibodies could also target sporozoites during the earlier stages of infection in the skin and circulation, and mediate effector functions to inhibit or kill sporozoites prior to reaching the liver.

We recently identified that antibodies can fix and activate the classical complement pathway against *P. falciparum* sporozoites, resulting in parasite lysis (21, 22). Functional complement-fixing antibodies were associated with protection against malaria acquired through natural exposure in a cohort of children, and were induced by the RTS,S vaccine in malaria-naïve adults and young children in a malaria endemic region, and may contribute to vaccine efficacy (22–24). Complement-fixation and cell killing can occur rapidly within minutes (25, 26) and is therefore a plausible mechanisms in immunity against sporozoites, and the importance of complement in antibody-mediated immunity has also been demonstrated in a mouse malaria model (27). Exploiting this effector function in vaccine development has the potential to target and clear sporozoites soon after inoculation and prevent sporozoites reaching the liver. Of further interest is that direct invasion-inhibitory activity of anti-CSP antibodies is strain-specific; some data suggest that complement-fixation may help negate the limitation of strain-specific activity (22). Emerging evidence points to an important role for antibody-complement interactions in immunity to malaria merozoite (25, 28) and gametocyte (29) forms, and against *P. falciparum* infected erythrocytes (30), and it is also important in some viral and bacterial infections (31).

Given the potential importance of complement-fixation by antibodies, it is important to further define the specific regions of CSP targeted and the antibody properties required for maximal function. Malaria immunity studies have often evaluated antibodies to the central-repeat region, which is considered immunodominant (9). Although, antibodies to the non-repeat regions of CSP may also be important in immunity and could target sporozoites during different stages of infection. Furthermore, antibody specificity and epitope repertoire can play a crucial role in mediating functional responses, as multiple IgG molecules are required to effectively fix the complement initiation protein C1q and activate the classical complement pathway (32, 33). Indeed, monoclonal antibodies to different epitopes of the same non-malaria antigen have demonstrated striking differences in the ability to activate complement (34).

Here, we investigated the specificities and properties of functional anti-CSP antibodies that fix C1q using antigen constructs representing the N-terminal, central-repeat, and C-terminal regions of the CSP. We evaluated the specificity and breadth of naturally acquired antibodies in Kenyan adults in relation to their ability to fix complement, and we evaluated the ability of rabbit antibodies generated to CSP and region-specific antigens to fix complement.

## Materials And Methods

### Human Study Participants and Ethics Approval

This study used plasma samples collected from a cohort study of adults resident in the Nyanza Province, Kenya (n=102), where malaria was holoendemic at the time (35). Participants were aged between 18 and 79 years. Plasma samples were collected in August, September and October of 2004, and samples collected at the September timepoint were used in this study. All participants gave written informed consent and ethics approval was obtained from the Institutional Review Board of Human Investigation, University Hospitals of Cleveland for Case Western Reserve University, United States of America (protocol 02-04-04); the Ethical Review Committee, Kenya Medical Research Institute, Kenya (protocol SSC867); and the Alfred Human Research and Ethics Committee, Australia (protocol 385-18). Prior to use, samples were heat-treated at 56°C for 45 minutes to inactive host complement proteins.

### Antigens

The following antigens used in this study were all based on *P. falciparum* 3D7 sequence (summarized in **Table 1** and illustrated in **Figures 1B, C**). We measured antibody responses to recombinant CSP using two similar constructs expressed in *Escherichia coli* (Gennova, India) or *Pichia pastoris* (Sanaria, USA) for human and animal studies, respectively. These were considered as near full-length CSP, as they lacked the signal peptide sequence at the N-terminus and the glycosylphosphatidylinositol sequence at the C-terminus (16, 36). Furthermore, the Sanaria CSP began at amino acid position 50 and contained 22 NANP major repeats and 4 NVDP minor repeats. We also examined antibody responses to the specific regions of CSP using several antigen constructs. These included synthetic peptides representing the N-terminal region between amino acids 19-104 (NT) (38) and the central-repeat region comprised of the NANP motif repeated 15 times (NANP), both synthesized and purified by Life Tein (USA). We also used a recombinant protein representing the C-terminal region between amino acids 237-383 (CT) and a truncated CSP that lacked the central-repeat region (N+C), both expressed in HEK293 cells as previously described (23, 38).

**Table 1:** Summary of *P. falciparum* antigens used in the study based on the 3D7 strain.

Antigen	Origin	Description/Sequence	Ref.
CSP (Gennova)	*E. coli*	Begins and ends at amino acids 19 and 383 of CSP, respectively, and contains the major NANP motif repeated 37 times	(36)
CSP (Sanaria)	*P. pastoris*	Begins and ends at amino acids 50 and 384 of CSP, respectively, and contains the major NANP motif repeated 22 times and the minor NVDP motif repeated 4 times	(16, 37)
NT (Life Tein)	Peptide	Amino acids 19-104 of CSP whereby the Cys^25^ was modified to Ser^25^. Sequence: LFQEYQSYGSSSNTRVLNELNYDNAGTNLYNELEMNYYGKQENWYSLKKNSRSLGENDDGNNEDNEKLRKPKHKKLKQPADGNPDP	(38)
NANP (Life Tein)	Peptide	Major NANP motif repeated 15 times. Sequence: NANPNANPNANPNANPNANPNANPNANPNANPNANPNANPNANPNANPNANPNANPNANP	(23)
CT (in-house)	HEK293	Amino acids 237-383 of CSP. Sequence: NKNNQGNGQGHNMPNDPNRNVDENANANSAVKNNNNEEPSDKHIKEYLNKIQNSLSTEWSPCSVTCGNGIQVRIKPGSANKPK DELDYANDIEKKICKMEKCSSVFNVVNSGS	(23)
N+C (in-house)	HEK293	Amino acids 26-104 and 273-383 of CSP (as above) fused with GGGS linker	(38)

### Generation of Antigen-Specific Antibodies in Rabbits

We generated rabbit IgG raised against the Sanaria near full-length CSP (200 µg/dose), the truncated N+C construct (200 µg/dose), and the NT (100 µg/dose) and CT (150 µg/dose) regions of CSP. Briefly, New Zealand White rabbits received three antigen immunizations each administered four weeks apart; the first dose was prepared in complete Freud’s adjuvant and the second and third dose in incomplete Freud’s adjuvant. Rabbit polyclonal IgG was purified from antisera collected 12 days after the final immunization for experimental use. Two rabbits were vaccinated with the CSP, N+C and CT antigens and because reactivity was comparable, we focused on testing purified IgG from a single rabbit in this study (**Figure S1A**). Two rabbits were also vaccinated with the NT antigen, but only one had high IgG reactivity by ELISA and was included in the study. Animal immunizations were conducted at the Animal Facility at the Walter and Eliza Hall Institute and ethics approval was obtained from the Animal Ethics Committee of the Walter and Eliza Hall Institute (ethics approval number: 2020.019).

### Antibody Responses by Standard Enzyme-Linked Immunosorbent Assay (ELISA)

ELISA was performed as previously described (22). Briefly, ninety-six well MaxiSorp flat bottom plates (Thermo Fischer Scientific, USA) were coated in 0.5 µg/ml antigen in phosphate buffered saline (PBS) overnight at 4°C. The following day, plates were blocked in 1% casein in PBS (w/v) and then incubated with test antibody samples. Human plasma samples were tested at 1/1000 dilution in buffer (0.1% casein in PBS, w/v), and antigen-specific antibodies were detected using goat anti-human IgG (Invitrogen, USA) and goat anti-human IgM (Millipore, USA) antibodies conjugated to HRP. Human plasma samples were also tested at 1/500 dilution for IgG subclass detection using mouse anti-human IgG1/IgG3 (Invitrogen), followed by goat anti-mouse IgG conjugated to HRP (Millipore). Purified rabbit IgG were tested at 0.001-10 µg/ml and detected using goat anti-rabbit IgG conjugated to HRP (Millipore). After the addition of HRP, plates were incubated with ABTS substrate (to detect human IgM and rabbit IgG) or TMB substrate (to detect human IgG and IgG subclasses) and absorbance was measured at an optical density (OD) of 405 nm and 450 nm, respectively. Note that all antibodies were prepared in 0.1% casein in PBS and plates were washed thrice using PBS-Tween20 0.05% between each incubation step.

### Complement-Fixation Assay

Ninety-six well plates were coated and blocked as described above for standard ELISA. Human plasma samples were tested at 1/100 dilution and purified rabbit IgG were tested at 0.1-1000 µg/ml. Plates were then incubated with purified human C1q (Millipore) at 10 µg/ml, and complement-fixation was detected using an in-house rabbit anti-C1q IgG for human samples (which has been previously validated) (23, 28) and goat anti-C1q (MP Biomedicals, USA) for rabbit samples, followed by species specific anti-IgG HRP conjugated antibodies (Millipore). After the addition of HRP, plates were incubated with TMB substrate and absorbance was measured at 450 nm. Note that antibodies and C1q were prepared in buffer and plates were washed thrice using PBS-Tween20 0.05% between each incubation step. We have previously shown that using purified C1q gives very similar results to using whole fresh serum as the source of complement and that C1q-fixation strongly correlates with downstream fixation of other complement components (22, 23, 28).

### Competition Assays

To confirm that antibodies targeting specific regions of CSP were involved in C1q-fixation, we also performed competition assays using established methods (39, 40). Briefly, the standard procedure to measure complement-fixation was followed, but antibody samples were pre-incubated with 10 μg/ml of competing antigen for 30 minutes prior to plate application.

### Statistical Analysis

Human plasma samples were tested in duplicate (mean of duplicates is shown) and corrected for background reactivity using no-plasma control wells and adjusted for plate-to-plate variation using positive control samples. The positivity threshold was calculated as the mean + 3 standard deviations of malaria-naïve negative controls from Melbourne donors. The median and interquartile range (IQR) were presented, and antibody reactivity between individuals who were positive or negative for C1q-fixation was compared using the Mann-Whitney U test (GraphPad Prism 8). We additionally performed logistic regression analysis to evaluate the relationship between antibody isotype (IgG and IgM) positivity and C1q-fixation positivity (StataSE 15) and the odds ratio (OR) was shown.

The rabbit IgG were tested in duplicate and corrected for background reactivity using no-IgG control wells. All experiments testing the purified rabbit IgG were performed twice, and the mean and range of two independent experiments is shown.

## Results

### Naturally Acquired Antibodies That Fix Complement Recognize All Three Regions of CSP

**Figure 2:**
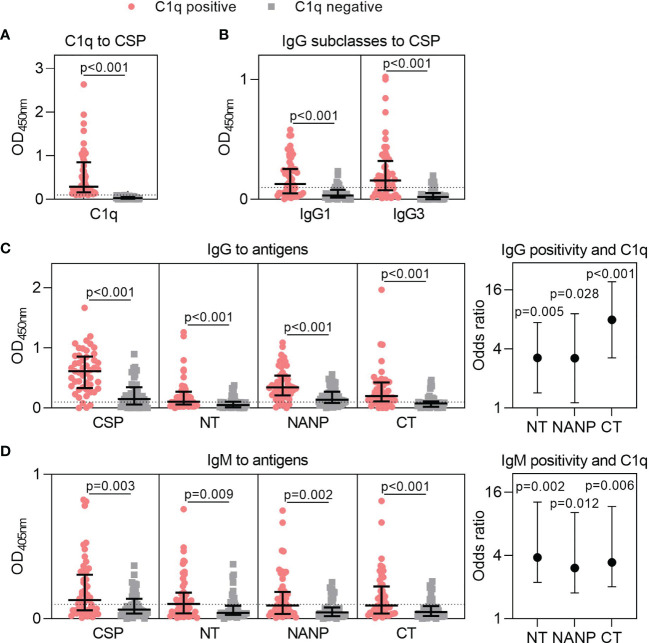
Targets of naturally acquired antibodies associated with complement-fixation to CSP. Plasma collected from malaria-exposed adults (n=102) were tested for **(A)** C1q-fixation to CSP and stratified into positive and negative groups (C1q positive, n=52; C1q negative, n=50). The C1q positive and C1q negative groups were tested for **(B)** IgG1 and IgG3 subclasses to CSP, and **(C)** IgG and **(D)** IgM to full-length CSP and antigens representing the N-terminal (NT), central-repeat (NANP) and C-terminal (CT) regions of CSP. Samples were tested in duplicate, and the mean of duplicates is shown along with the group median and IQR. Dotted lines represent the positivity threshold relative to malaria-naïve negative controls. Reactivity between C1q positive and C1q negative groups were compared using the Mann-Whitney U test. In panels **(C, D)**, the odds ratio and 95% confidence interval are shown for IgG/IgM positivity to each region of CSP and C1q-fixation positivity using logistic regression; y-axis was presented on a log2 scale.

We previously established that human antibodies can fix C1q and activate the classical complement pathway (22, 25). Here, we tested plasma samples from Kenyan adults naturally exposed to malaria (n=102) and found that 51% of individuals had antibodies that could fix C1q to CSP [OD median (IQR): C1q positive = 0.289 (0.162, 0.847); C1q negative = 0.030 (0.015, 0.059), **Figure 2A**]. To better understand this variable acquisition of functional antibodies, we next quantified IgG responses. This included the IgG1 and IgG3 subclasses to CSP, which were significantly higher for individuals defined as positive for C1q-fixation activity (p<0.001 for both tests, **Figure 2B**). We next evaluated total IgG against CSP and region-specific antigens representing the N-terminal (NT), central-repeat (NANP) and C-terminal (CT) regions of the protein. IgG to near full-length CSP was significantly higher among C1q positive individuals than C1q negative individuals [OD median (IQR): C1q positive = 0.613 (0.332, 0.857); C1q negative = 0.149 (0.059, 0.347); p<0.001, **Figure 2C**]. This was expected as antibody concentration is known to influence complement-fixation. However, we also observed that IgG to all three region-specific antigens were significantly higher in participants positive for C1q-fixing antibodies than those who were negative (p<0.001 for all tests, **Figure 2C**).

We additionally measured IgM to CSP and the three region-specific antigens. Individuals who were positive for C1q-fixation had significantly higher IgM to all antigens compared to those who were negative for C1q-fixation (p<0.010 for all tests, **Figure 2D**). Collectively, these data suggest that increased IgG and IgM to all three regions of CSP contribute to functional complement-fixing responses, as confirmed by logistic regression analysis (OR≥3.039 and p<0.05 for all tests, **Figures 2C, D**).

### Antibody Breadth Is Associated With Complement-Fixation

**Figure 3:**
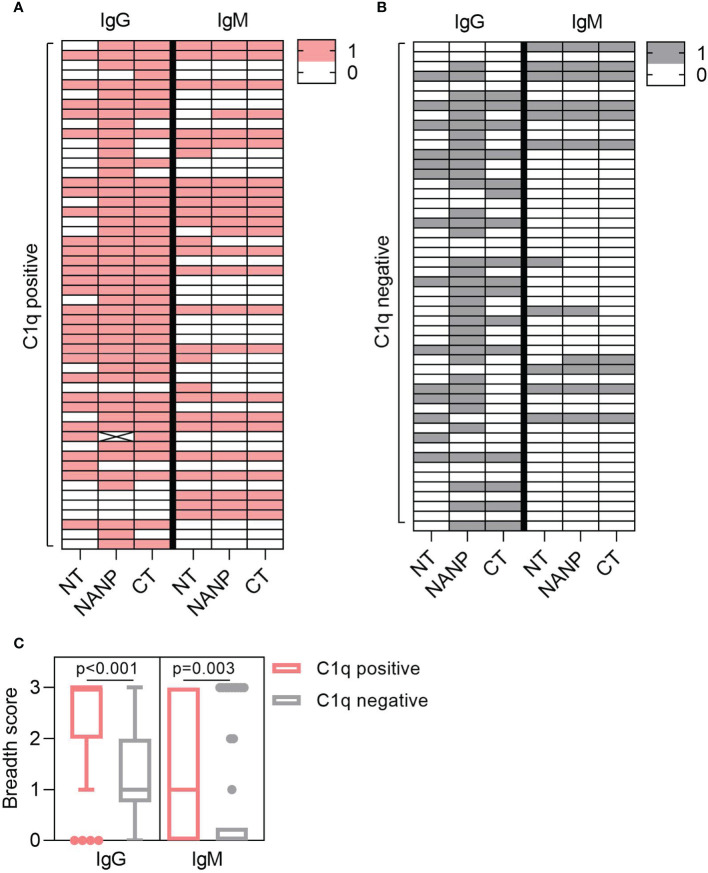
Antibody breadth is associated with C1q-fixation. Plasma collected from malaria exposed adults (n=102) were tested for C1q-fixation to CSP and stratified into **(A)** C1q positive (n=52) and **(B)** C1q negative (n=50) groups. Heat-maps display IgG/IgM seropositivity to antigens representing the N-terminal (NT), central-repeat (NANP) and C-terminal (CT) regions of CSP. Each row represents an individual participant that was defined as seropositive (score = 1) or seronegative (score = 0) for IgG or IgM to each antigen. **(C)** The breadth scores for IgG and IgM were calculated by combining the seropositivity score for all three antigens (total breadth scores were between 0 and 3). The IgG and IgM breadth score for the C1q positive and C1q negative groups are shown as box plots (box represents the 25th and 75th percentile, whiskers represent the highest and lowest values within 1.5xIQR and values outside this range are shown as dots) and were compared using the Mann-Whitney U test.

Complement-fixation was associated with increased antibodies to each region of CSP, but antibody breadth may also play a role. To explore this, we determined the antibody breadth score. Participants were defined as seropositive (score = 1) or seronegative (score = 0) for antibody responses to each region-specific antigen (NT, NANP, CT), presented as heat-maps (**Figures 3A, B**). These scores were combined to give the overall breadth score, whereby a score of 0 meant the individual was seronegative for all antigens and a score of 3 meant the individual was seropositive for all antigens. Breadth scores were calculated for IgG and IgM and compared between individuals that were positive or negative for C1q-fixation. The breadth score of IgG and IgM were significantly higher in the C1q positive group compared to the C1q negative group (IgG, p<0.001; IgM, p=0.003, **Figure 3C**).

### Targets of Functional Antibodies Generated Against CSP in Rabbits

**Figure 4:**
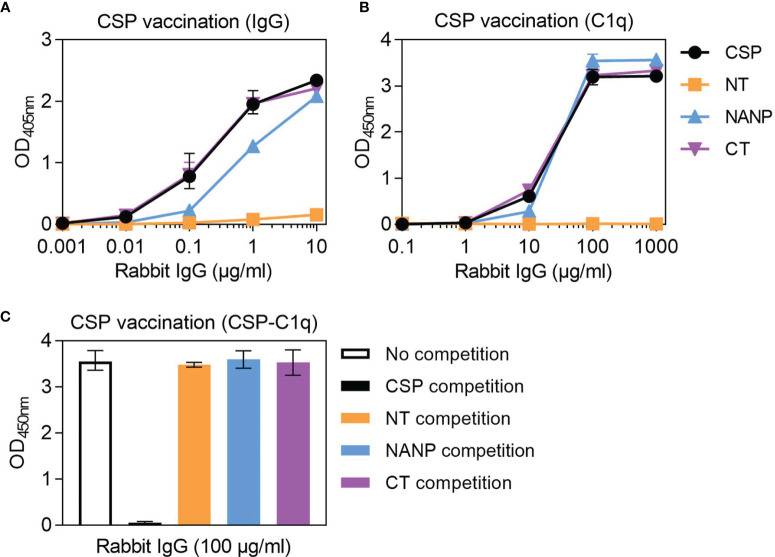
Complement-fixing activity of rabbit anti-CSP IgG to different regions of CSP. Rabbit anti-CSP IgG was tested for **(A)** IgG at 0.001-10 μg/ml and **(B)** C1q-fixation at 0.1-1000 μg/ml to CSP and antigens representing the N-terminal (NT), central-repeat (NANP) and C-terminal (CT) regions of CSP; x-axis was presented on a log10 scale. **(C)** The rabbit anti-CSP IgG was also tested at 100 μg/ml for C1q-fixation to CSP after pre-incubation with competing anitgen (including a no competition control); see **Figure S1B** for related data. Results were corrected for background reactivity using no-IgG control wells and the mean and range of two independent experiments is shown.

To act as a model for vaccination, we generated rabbit antibodies against CSP, as rabbit IgG is similar to human IgG1 and can fix and activate human complement (41). The rabbit anti-CSP IgG had strong reactivity to CSP (**Figure 4A**). There was also strong reactivity to the NANP and CT antigens, but poor reactivity to the NT antigen, even at the highest concentration tested (10 μg/ml). The rabbit anti-CSP IgG could fix C1q to full-length CSP, NANP and CT, but not to the NT antigen (**Figure 4B**).

To further explore the role of antibody specificity in complement-fixation responses, we used competition assays and confirmed that 10 μg/ml of competing antigen was sufficient for complete homologous competition (**Figure S1B**). In these experiments, the rabbit anti-CSP IgG was pre-incubated with competing antigen (NT, NANP or CT) and then tested for C1q-fixation against CSP (**Figure 4C**). Competing NT had no effect on C1q-fixation to CSP, which was expected given that the rabbit antibody poorly recognized this antigen. However, competing NANP and competing CT also had no effect on C1q-fixation. These data suggest that the central-repeat and C-terminal regions of CSP are both targets of complement-fixing antibodies, and that antibody binding to only one of these regions was sufficient to promote C1q-fixation against CSP.

### Antibodies to Non-Repeat Regions of CSP Can Strongly Fix Complement

**Figure 5:**
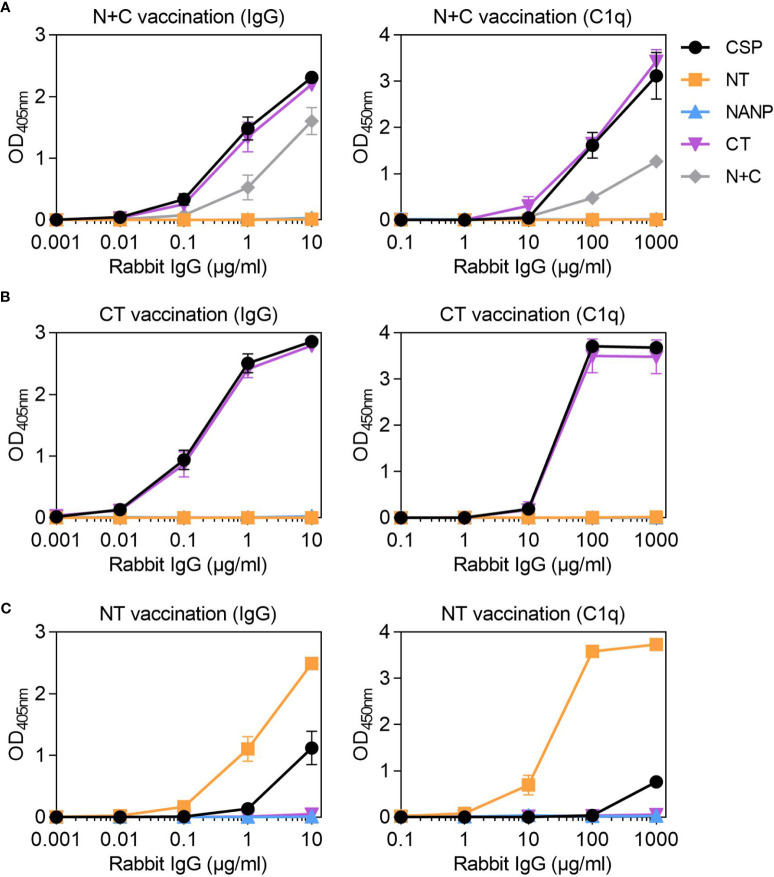
Rabbit IgG to the non-repeat regions of CSP can fix complement. Rabbit IgG were generated against **(A)** truncted CSP that lacked the central-repeat region (N+C) and the **(B)** C-terminal (CT) and **(C)** N-terminal (NT) regions of CSP. Rabbit IgG were tested for IgG at 0.001-10 μg/ml (left) and C1q-fixation at 0.1-1000 μg/ml (right) against full-length CSP, NT, NANP and CT anitgens [and N+C for panel **(A)**]; x-axis was presented on a log10 scale. Results were corrected for background reactivity using no-IgG control wells and the mean and range of two independent experiments is shown.

Studies of humoral immunity have largely focused on the central-repeat region of CSP and can overlook the potential role of non-repeat antibodies in immunity. Considering humans naturally acquired antibodies to the repeat and non-repeat regions of CSP, we further explored the latter in rabbit immunization studies. We generated rabbit antibodies against a truncated form of CSP that lacked the central-repeat region, referred to as N+C. The rabbit anti-N+C IgG had strong IgG reactivity to CSP and CT, and slightly lower reactivity to the N+C construct (**Figure 5A**). There was no antibody binding to NANP, indicating we had successfully generated antibodies to the non-repeat regions of CSP; however, there was also little binding to the NT antigen. The rabbit anti-N+C IgG could strongly fix C1q to CSP and CT antigens, and less strongly to N+C (**Figure 5A**). To confirm the role of CT-specific antibodies in C1q-fixation, we performed another rabbit immunization with the CT antigen alone. The rabbit anti-CT IgG had strong IgG reactivity against CSP and CT, with no reactivity against the NT and NANP antigens (**Figure 5B**). These antibodies could also fix C1q to full-length CSP and CT (**Figure 5B**).

Since vaccination with the near full length or truncated CSP constructs did not effectively generate antibodies to NT, we vaccinated rabbits with the peptide representing just the NT region. The rabbit anti-NT IgG demonstrated strong IgG binding to the NT antigen, which confirmed that antibodies to this region could be induced by vaccination. However, there was much lower IgG binding to CSP (**Figure 5C**). These antibodies could strongly fix C1q to NT, but activity to near full-length CSP was weak (**Figure 5C**). Given that rabbits vaccinated with CSP poorly induced antibodies to the N-terminal region of the protein, it was of interest to assess whether this occurred other animal models. We have previously shown that mice (n=5) vaccinated with the near full-length Gennova CSP formulated with an alum adjuvant generated IgG to the NANP and CT antigens. Here, we tested the mouse antiserum for IgG to the NT antigen, and similarly found poor antibody reactivity to the N-terminal region of CSP (**Figure S1C**).

Overall, we were able to generate rabbit antibodies to the N-terminal and C-terminal regions of CSP, which were also confirmed to recognize *P. falciparum* sporozoites by ELISA (**Figure S1D**). These data demonstrate that non-repeat antibodies, mostly targeting the C-terminal region, can sufficiently promote C1q-fixation to full-length CSP.

## Discussion

Identifying the targets of functional complement-fixing antibodies can provide valuable insights to improve leading malaria vaccine candidates or to develop novel vaccine constructs. Here, we investigated the N-terminal, central-repeat, and C-terminal regions of CSP as targets of complement-fixing antibodies acquired by natural malaria exposure in humans and induced by vaccination in rabbits, as well as human antibody types mediating complement-fixation. Firstly, we evaluated a cohort of Kenyan adults and found that 51% of individuals had detectable C1q-fixing antibodies. These individuals had significantly higher IgG and IgM to all three regions of CSP and higher antibody breadth to these regions compared to those defined as negative for C1q-fixation. Secondly, we generated rabbit anti-CSP IgG to evaluate the role of region-specific antibodies in mediating complement-fixation. Using competition assays, we confirmed that antibodies targeting the central-repeat or C-terminal regions alone could effectively mediate C1q-fixation to full length CSP. Notably, vaccination with near full-length CSP poorly generated antibodies to the NT region. Antibodies to the N-terminal region were only strongly induced by vaccination with the NT antigen alone, which demonstrated some complement-fixing activity.

Higher anti-CSP IgG and IgM were detected in Kenyan adults positive for C1q-fixing antibodies compared to individuals who were negative for functional responses. This was expected as IgG (specifically the IgG1 and IgG3 subclasses) and IgM can fix C1q and activate the classical complement pathway, and we recently found that complement activation by IgM acquired to malaria antigens had greater complement activity (42). We have previously demonstrated that C1q-fixation by anti-CSP antibodies correlates strongly with subsequent complement activation, leading to formation of the membrane attack complex (22). There is likely an antibody threshold required to mediate C1q-fixation, although it is difficult to infer this threshold given that we evaluated polyclonal antibodies in plasma samples, including a mixture of different antibody isotypes, IgG subclasses, and antibodies with different epitope specificities. While antibody threshold is one factor that contributes to antibody functionality, our data demonstrates that fine specificity and breadth also are also important, and often overlooked in immunogenicity studies. C1q positive individuals had higher IgG and IgM specific to the central-repeat region, which is considered immunodominant and given its long, repetitive and flexible nature, seems a likely target involved in antibody-mediated complement-fixation (9). Indeed, it has been shown that only 2.5 repeats are needed for antibody binding, which could therefore accommodate multiple IgG molecules needed for C1q-fixation (43).

We showed that antibodies are also acquired through natural malaria exposure to the N-terminal and C-terminal regions of CSP, which are understudied targets of immunity. There was a previous report that antibody samples from Tanzanian children recognized a peptide representing the N-terminal region (amino acids 65-110) and responses were associated with protection against clinical malaria (44). However, the peptide used in that study included the NANPNV sequence, part of the minor repeat sequence that is known to be a target of antibodies (45). We synthesized the same peptide and found that it could also be recognized by a repeat-specific monoclonal antibody, possibly due to the NANPNV sequence (**Figure S2**). It was therefore unclear whether that study had measured antibodies to the N-terminal region alone, or antibodies to the central-repeat region that cross-reacted with the peptide used. Another study had evaluated natural antibody responses to an N-terminal construct that also overlapped with the central-repeat region (amino acids 16-122) and several C-terminal constructs, although these represented smaller fragments of the C-terminal and not the entire region (46). Therefore, there has been limited data available on naturally-acquired antibodies specific to the N-terminal and C-terminal regions of CSP.

In our study, antibodies to the non-repeat regions of CSP were significantly higher in individuals with complement-fixing antibodies compared to negative responders, highlighting their potential role in functional immunity. This may be particularly important as the CSP has different conformations whereby the N-terminal region may partially mask the TSR region (within the C-terminal region) but is then cleaved prior to hepatocyte invasion (12, 13). Therefore, antibodies to the N-terminal region may target sporozoites in the skin and circulation, while antibodies to the C-terminal region may target, at least partly, sporozoites prior to invasion. *In vitro* studies have shown that antibodies to the non-repeat regions can recognize *P. falciparum* sporozoites and inhibit migration and cellular invasion (7, 12, 47–49). Perhaps these functions could be further enhanced by complement, since other studies of anti-sporozoite immunity have shown that complement can enhance inhibition of sporozoite motility and invasion by antibodies (21, 22). Furthermore, recent studies reported that antibodies to the NT and CT regions can promote opsonic phagocytosis of sporozoites, primarily by neutrophils (38), indicating that antibodies to the non-repeat regions have multiple functional activities. We also found that antibody breadth to multiple regions of CSP was higher among individuals positive for complement-fixation. This suggests that antibodies to multiple regions may have a synergistic effect to mediate complement-fixation, which has been seen for other non-malarial antigens (50). Together, our human data show that antibodies to the repeat and non-repeat regions of CSP are naturally acquired and appear important in mediating complement-fixation.

To further understand the role of antibody specificity in mediating complement-fixation, we generated region-specific antibodies in rabbit vaccination studies. Rabbits were selected as rabbit IgG can strongly activate human complement and are suitable for obtaining large quantities of IgG for experimental use. We firstly generated rabbit IgG against near full-length CSP, which strongly recognized the NANP and CT antigens. The rabbit anti-CSP IgG could mediate C1q-fixation to wells directly coated in NANP and CT antigens, suggesting these were each important targets of functional antibodies. We also used competition assays to confirm that antibodies to the central-repeat or C-terminal regions alone were strongly capable of mediating C1q-fixation to CSP. It would be valuable to explore these responses further using whole sporozoites in future studies.

Notably, vaccination with near full-length CSP or a truncated form that lacked the central-repeat region were both unable to induce antibodies to the N-terminal region of the protein. This was in striking contrast to the human study whereby natural malaria exposure induced antibodies to the N-terminal region. The reasons for this were unclear; perhaps it was due to the conformation of the antigen constructs used. It was also possible that vaccination with the Sanaria CSP poorly induced antibodies to the NT region, as this protein had some truncation of sequence at the N and C-terminus and began at amino acid 50. However, this seems unlikely, as the N+C construct also poorly induced antibodies to the NT region and began at amino acid 19, as did vaccination with the Gennova near full-length CSP in mice. A likely reason for the low activity of rabbit antibodies to the NT region is that the N-terminal region was poorly immunogenic, or that the other regions were immunodominant. This was supported by the fact that we were able to induce these antibodies by vaccination with the NT antigen alone. This may have important implications for vaccine development as vaccines based on full-length CSP may not be effective in generating antibodies to the NT region. There is a question of whether the N-terminal region should be included in the RTS,S vaccine for example, which currently only contains the central-repeat and C-terminal regions. Antibodies to additional epitopes may be generated by the inclusion of N-terminal epitopes or the junction epitope that may be important for functional immunity (45, 51).

Once we had generated rabbit antibodies specific to the N-terminal and C-terminal regions, we confirmed that both could fix complement to CSP, although responses were lower for the former. Antibodies specific to the repeat and non-repeat regions mediate complement-fixation to full-length CSP. At this point, we cannot draw any conclusions on whether one region was more immunogenic than another. It would be valuable for future studies to examine the specific epitopes within each region, or combinations of epitopes that may have synergy and promote enhanced complement-fixation. Such studies may inform future vaccine design. It may also be valuable to study the induction and role of IgM in future rabbit immunogenicity studies, given the importance of acquired IgM identified in our human study.

Our data highlight an important role for the repeat and non-repeat regions of CSP as targets of functional complement-fixing antibody responses. Humans naturally exposed to malaria acquired IgG and IgM responses to all three regions of the CSP. Greater antibody breadth to these regions appeared important for mediating complement-fixation, and IgG1, IgG3 and IgM were associated with complement-fixation. In a series of rabbit vaccination studies, we demonstrate that antibodies to the repeat and non-repeat regions can directly mediate complement fixation to full-length CSP. Notably, we observed major differences in antibody responses to the N-terminal region of CSP between human immunity from natural malaria exposure versus vaccine-induced antibodies in animal studies that warrants further investigation. Redesign of vaccine constructs to maximize the induction of complement-fixing antibodies may be a strategy to achieve higher vaccine efficacy. Our findings suggest vaccines that generate antibodies with broad activity to all three regions of CSP may be most effective and indicate roles for both IgG and IgM.

## Data Availability Statement

All data are available from the authors on reasonable request, and dependent on ethics and regulatory approval (for human data).

## Ethics Statement

The studies involving human participants were reviewed and approved by Institutional Review Board of Human Investigation, University Hospitals of Cleveland for Case Western Reserve University, United States of America (USA), Ethical Review Committee, Kenya Medical Research Institute, Kenya, the Alfred Human Research and Ethics Committee, Melbourne, Australia. The patients/participants provided their written informed consent to participate in this study. The animal study was reviewed and approved by Animal Ethics Committee of the Walter and Eliza Hall Institute.

## Author Contributions

LK performed all experiments and data analysis. DD expressed recombinant proteins used in the study. AD and JK were involved in the Kenyan cohort study. LK and JB designed the study and wrote the manuscript. All authors contributed to the article and approved the submitted version.

## Funding

This work was funded by the National Health and Medical Research Council (NHMRC) of Australia (Program Grant 1092789, Project Grant 1141278, and Investigator Grant 1173046 to JB) and the Australian Government Research training Program Scholarship to LK, and the NIH International Centers for Excellence in Malaria Research (U19 AI129326).

## Conflict of Interest

The authors declare that the research was conducted in the absence of any commercial or financial relationships that could be construed as a potential conflict of interest.

## Publisher’s Note

All claims expressed in this article are solely those of the authors and do not necessarily represent those of their affiliated organizations, or those of the publisher, the editors and the reviewers. Any product that may be evaluated in this article, or claim that may be made by its manufacturer, is not guaranteed or endorsed by the publisher.
