# Supplementary material for: Antibody Targets and Properties for Complement-Fixation Against the Circumsporozoite Protein in Malaria Immunity
Source: Front Immunol. 2021 Dec 1;12:775659. doi: 10.3389/fimmu.2021.775659 (PMC8671933; doi:10.3389/fimmu.2021.775659)
Supplement: Supplementary file 1 [file DataSheet_1.docx]

## **Supplementary:** Antibody targets and properties for complement fixation against the circumsporozoite protein in malaria immunity

## AUTHORS AND AFFILIATIONS

Liriye Kurtovic^1, 2^, Damien R. Drew^1^, Arlene E. Dent^3^, James W. Kazura^3^, James G. Beeson^1, 2, 4, 5*^

^1^Burnet Institute, Melbourne, Australia.

^2^Department of Immunology and Pathology, Monash University, Melbourne, Australia.

^3^Center for Global Health and Diseases, Case Western University, Cleveland, USA.

^4^Department of Microbiology, Monash University, Clayton, Australia.

^5^Department of Medicine, The University of Melbourne, Parkville, Australia.

*Corresponding author: 85 Commercial Road, Melbourne, Australia, 9282 2111, beeson@burnet.edu.au

#

## SUPPLEMENTARY METHODS

### Mouse anti-CSP vaccination study

We previously showed that mice (n=5) vaccinated with near full-length Gennova CSP in an alum adjuvant induced IgG to the central-repeat and C-terminal regions of CSP ([1](#_ENREF_1)). Here, we tested the mouse antisera at 1/2000 dilution for IgG to the NT antigen that represented the N-terminal region of CSP (**Figure S2c**). The mouse antisera were tested by ELISA as described in the main results section and was detected using goat anti-mouse IgG HRP conjugated antibodies (Millipore).

### Sporozoite ELISA

Rabbit IgG were tested for reactivity against *P. falciparum* sporozoites as described for standard ELISA, coating at 10,000 cells per well and all washes were performed using PBS (**Figure S1b**). Cryopreserved *P. falciparum* 3D7 sporozoites (Sanaria, Rockville, USA) were provided by PATH’s MVI.

### N-terminal study using a 46 amino acid peptide

A previous study reported that antibody responses to a 46 amino acid peptide representing the N-terminal region of CSP were associated with clinical protection. We synthesized the same peptide (LKKNSRSLGENDDGNNEDNEKLRKPKHKKLKQPADGNPDPNANPNV; Life Tein, USA) and found that it could be recognized by human monoclonal antibodies (mAbs) specific to the N-terminal and central-repeat regions of CSP (mAbs were provided by PATH’s MVI, **Figure S2**). This was likely due to the repeat-specific mAb cross-reacting with the terminal NANPNV sequence of the 46 amino acid peptide.


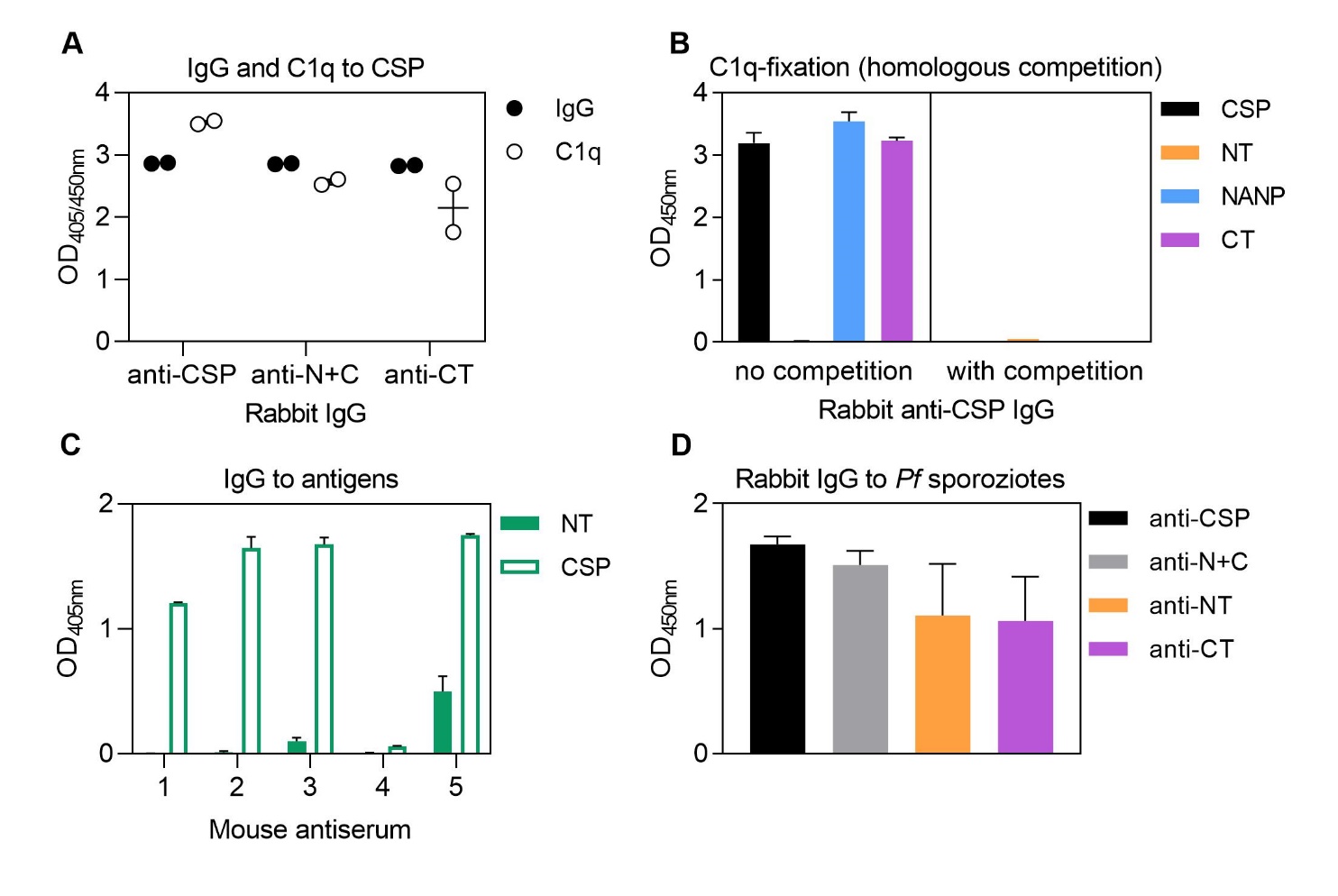


**FIGURE S1. Supplementary rabbit and mouse antibody data. A**) Rabbits (n=2) were vaccinated with CSP, N+C and CT, and purified IgG (from antiserum collected after vaccination) were tested for IgG and C1q-fixation to CSP. Results were corrected for background reactivity using no-IgG control wells and each symbol represents a rabbit, and the mean and range of rabbits is shown. **B**) Rabbit anti-CSP IgG was tested for C1q-fixation to CSP and antigens representing the N-terminal (NT), central-repeat (NANP) and C-terminal (CT) regions of CSP. The rabbit anti-CSP IgG was tested against each antigen following pre-incubation with 10 μg/ml of homologous competing anitgen, or no competition as a control. Results were corrected for background reactivity using no-IgG control wells, and the mean and range of two independent experiments is shown. **C**) Mice (n=5) were previously vaccinated with CSP and the antiserum was tested for IgG to the NT anitgen. Results were corrected for background reactiviy using no-serum control wells and the mean from two independent expeirments is shown. Note that data on reactivity to CSP were previously published (Kurtovic, Wezel et al., Front Immunol 2021 ([1](#_ENREF_1))) and have been re-analyzed here for comparison. **D**) Rabbit IgG against CSP, N+C, NT and CT were tested for IgG to *P. falciaparum* sporozoites. Results were corrected for background reactivity using no-IgG control wells, and the mean and range is shown.


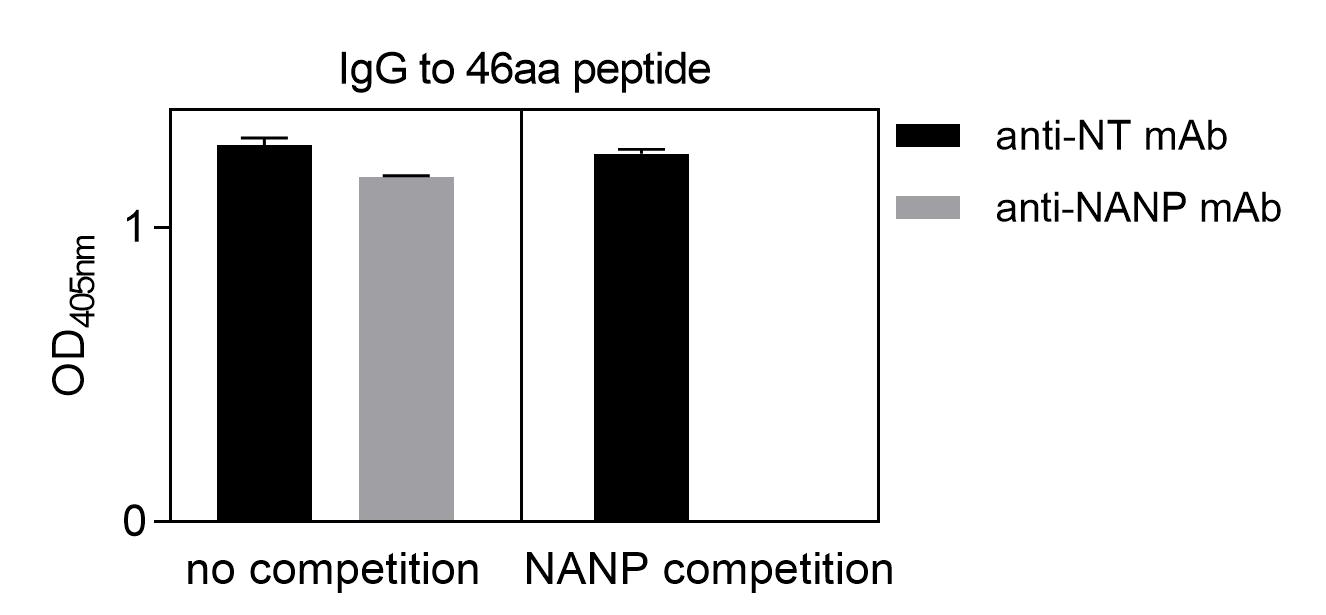


**FIGURE S2. Supplemtary data using a 46 amino acid peptide representing the N-terminal region of CSP**. Human anti-NT and anti-NANP mAbs were tested for IgG reactivity against the NT 46 amino acid (aa) peptide in the presence and absence of competing NANP. Results were corrected for background reactivity using no-IgG control wells, and the mean and range were shown.

## SUPPLEMENTARY REFERENCES

1. Kurtovic L, Wetzel D, Reiling L, Drew D, Palmer C, Kouskousis B, et al. Novel virus-like particle encoding the circumsporozoite protein of *Plasmodium falciparum* induces functional antibody responses in mice. Front Immunol. 2021;12:788.
